# Prognostic impact of neurocognitive disorders in older patients with cancer: the ELCAPA prospective cohort study

**DOI:** 10.1016/j.jnha.2024.100215

**Published:** 2024-03-21

**Authors:** Catherine Conti, Elena Paillaud, Marie Laurent, Johanne Poisson, Pascaline Boudou-Rouquette, Maxime Frelaut, Pierre Gay, Johanna Canovas, Philippe Caillet, Soraya Mebarki, Amaury Broussier, Florence Canouï-Poitrine

**Affiliations:** a AP-HP, Paris Cancer Institute CARPEM, Hôpital Européen Georges Pompidou, Department of Geriatrics, F-75015 Paris, France; b Université Paris-Est, Inserm, 7376- IMRB, F-94000 Créteil, France; c AP-HP, Hopital Henri-Mondor, Department of Internal Medicine and Geriatrics, F-94010 Creteil, France; d Université Paris-Cité, Center for Research on Inflammation, Inserm U1149, F-75018 Paris, France; e AP-HP, Hôpital Cochin – Port-Royal, Department of Oncology, F-75014 Paris, France; f Gustave Roussy Cancer Campus, F-94076 Villejuif, France; g AP-HP, Hôpitaux Henri-Mondor/Emile Roux, Department of Geriatrics, F-94456 Limeil-Brevannes, F-94000 Créteil, France; h AP-HP, Hopital Henri-Mondor, Public Health Departement, F-94010 Creteil, France

**Keywords:** Neurocognitive disorder, Older adult, Cancer, Prognostic value, Mortality

## Abstract

**Objective:**

To assess the prognostic value of neurocognitive disorder (NCD) for 12 month-overall mortality in patients aged 70 or more with a solid cancer.

**Design:**

prospective, observational, multicenter cohort.

**Setting and participants:**

We analyzed data from the ELCAPA longitudinal multicenter observational cohort of patients aged 70 or over, referred for a geriatric assessment (GA) before a new cancer treatment modality between January 31st, 2007, and December 29th, 2017. We defined the baseline NCD in four classes: no NCD, mild NCD, moderate NCD, and major NCD, based on the Mini-Mental State Examination (MMSE) score, memory complaint, and the Instrumental Activities of Daily Living (IADL) score.

**Statistical methods:**

We compared the baseline characteristics of patients according to NCD classes, globally and by pairs (with Bonferroni’ correction). Prognosis value of NCD classes were analysed by using univariable and then multivariable 12 month survival analysis with age as time-variable and with and without adjustement for the treatment strategy (curative, palliative or exclusive supportive care).

**Results:**

2784 patients with solid-cancer were included, with a median [interquartile range] age of 82 [78;86]. 36% of the patients were free of NCD, 34% had a mild NCD, 17% had a moderate NCD, and 13% had a major NCD. We identified the following independent prognostic factors for 12 month-overall mortality: NCD (adjusted hazard ratio (aHR) [95% confidence interval (CI)] for a major NCD = 1.54 [1.19–1.98] (p < 0.001), type of cancer, metastatic status, inpatient consultation, poor general health (assessed as the level of fatigue and Eastern Cooperative Oncology Group performance status [ECOG-PS]), greater weight loss, palliative treatment, and exclusive supportive care. Additional adjustment for the treatment strategy did not greatly change the strength of the association of a major NCD with 12 month-overall mortality (HR [95%CI] = 1.78 [1.39–2.29] (p < 0.001).

**Conclusion:**

Our results suggest that the presence of a major NCD has direct prognostic value (independently of other geriatric factors, the type of cancer and the treatment strategy) in older patients with a solid cancer.

## Highlights

• In older patients with cancer neurocognitive disorder is more prevalent than in younger patients and may have an impact on the disease outcome.
• Major neurocognitive disorder was associated with higher overall mortality in older adults with cancer, independently of frailty and treatment.
• Prognosis and treatment strategies might be refined by a more precise assessment of neurocognitive disorder in older patients with solid cancer.

## 1 Introduction

It has been estimated that 4 million new cancers occurred in Europe in 2020, 60% of which occurred in patients over the age of 65. Seventy-three percent of cancer-related deaths occur in patients aged 65 or over [1]. Despite these statistics, older adults with cancer are still under-represented in clinical trials. This leads to uncertainty about the real-life risk-benefit ratio for treating these older patients [2,3].

A prognostic assessment is a major step in therapeutic decision-making in the field of cancer. It is more complicated in older adults, due to heterogeneity in comorbidities, cognitive and mood disorders, nutritional status, functional status, and the social environment. All of which are criteria for frailty [4]. In older adults in general, frailty criteria are more predictive than chronological age for mortality, loss of autonomy, hospital admission, physical limitations, falls, and fractures [5]. In older patients with cancer, frailty is additionally associated with an elevated risk of complications and adverse drug reactions [6]. In order to predict mortality and treatment toxicity [7,8], the current guidelines recommend the assessment of frailty criteria with a Geriatric Assessment (CGA) [9,10] in cancer patients aged 70 or over. This evaluation, prior to treatment initiation, includes evaluation of neurocognitive disorder (NCD).

Considering the multifaceted challenges of older adults, NCDs are emerging as a crucial factor in understanding disease outcomes. The coexistence of a NCD and cancer is more prevalent in older patients (from 0.2% to 45.6% [11]) than in younger patients. It may have an impact on the disease outcome. The presence of a NCD is an independent prognostic factor in older patients in general. It is subject to debate in the specific population of older patients with cancer [[12], [13], [14]]. Furthermore, the nature of the association (direct or indirect) has not been determined. NCD is often associated with other frailty criteria that accentuate cognitive impairment [15] - especially during or after chemotherapy [[16], [17], [18]].

However, studies studying the link between cognitive impairment and death in over-65 people without cancer showed that the strength of the association decreased when taken into account other comorbidities and/or physical frailty [19]. In addition, the choice of therapeutic strategies, which is more difficult and less standardized in older subjects with NCD, could be a mediating or a confounding factor in the NCD - mortality relationship [20]. Therefore, understanding the link between NCD and cancer treatment decision (e.g., more likely palliative than curative for example) is of importance to properly assess the prognosis value of NCD.

The main objective of the present study was to assess the prognostic value of NCDs for 1-year overall mortality in patients aged 70 or over with a solid cancer. The secondary objective was to assess the impact of NCDs on the cancer treatment decision.

## 2 Material and methods

We analyzed data from the Elderly Cancer Patient (ELCAPA) cohort study. This is a French, prospective, observational, multicenter cohort of patients aged 70 years or over with a newly diagnosed solid or hematological cancer at any stage and who had been referred to a geriatrician for a standardized CGA at one of 19 centers in the Ile-de-France region prior to treatment initiation.

The ELCAPA study protocol has been approved by an institutional review board (*CPP Ile-de-France I*, Paris, France; reference: 2019 mai-MS121) and registered on ClinicalTrials.gov (NCT:02884375). In line with the French legislation on this type of study, the provision of written, informed consent was not required.The patient is informed of the objectives and the modalities of the study through a detailed information sheet. This sheet also precised that the participation to this research is entirely free and voluntary and that the decision to participate or not do not affect the quality of care and treatment. The patient may withdraw from the research at any time without justification, and without any consequences for the continuation of the treatment or the quality of care provided to you, or relationship with the physician. The medical file will remain confidential and may only be consulted under the responsibility of the physician in charge of your treatment and by persons duly authorised for research purposes and subject to professional secret.

Here, we focused on patients included for CGA between January 31st, 2007, and December 29th, 2017. Follow-up was continued until December 31st, 2018. The main non-inclusion criteria for the present study were delirium, not speaking French, and hematological cancer. We did not include hematological cancer as the therapeutic strategy differ substantially from solid cancer.

### 2.1 Endpoints

The primary endpoint was death at 12 months after the CGA. The choice of 12-months as time frame was based on previous study in SEER-Medicare database showing that pre-existing dementia prior to diagnosis of solid cancer has an impact on survival from 6 months and is maximal at 1 year [21]. Vital status was collected by matching the ELCAPA cohort’s data with the French national vital statistics registry (*Répertoire National d'Identification des Personnes Physiques*, Paris, France). The secondary endpoint was the treatment strategy selected at the multidisciplinary team meeting involving surgeons, oncologists and pathologists based on the institutional guidelines or protocols and taking account of geriatric assessment. The treatment strategy has been classified as curative treatment if the goal was to achieve complete remission or palliative treatment when the goal was to reduce the cancer’s progression or exclusive supportive care intended to reduce patient’s symptomes.

### 2.2 Covariates

On inclusion, we collected data on sociodemographic variables (age and sex), the initial mode of care (inpatient or outpatient treatment), and oncological variables (primary tumor site and metastatic status). The geriatric assessment was performed on inclusion by a geriatrician with expertise in oncology. The following variables were documented: type of living environment (own home or in an institution), autonomy (the six-item activities of daily living [ADL] [22] score [abnormal if < 6], the four-item instrumental activities of daily living [IADL] [23] score [abnormal if <4]), risk of falling (including a history of falls in the previous 6 months and the one-leg standing balance test [24] [abnormal if the patient cannot stand on one leg for 5 s or longer]). Functional status was described with regard to the Eastern Cooperative Oncology Group Performance Status (ECOG-PS) scale [25] and categorized into three levels (overall health preserved: ECOG-PS 0−1, reduced physical activity: ECOG-PS 2, bedridden or in a chair >50% of the time: ECOG-PS 3−4) and the presence of fatigue. Nutritional status was defined according to the percentage weight loss: malnutrition for weight loss >5% in 1 month or >10% in 6 months, and severe malnutrition for weight loss >10% in 1 month or >15% in 6 months according to the 2007 Haute Autorité de Santé (Health Authority, HAS) guidelines. The comorbidities included probable depression (according to the Mini Geriatric Depression Scale (GdS) score [26]; abnormal: score ≥1), arterial hypertension, other cardiovascular diseases (rhythm disorders, heart failure, and coronary artery disease), chronic kidney disease (CKD) according to the 2012 Haute Autorité de Santé classification: "no CKD" if the glomerular filtration rate (estimated with the Cockcroft formula) is ≥60 ml/min, "mild CKD" for a value between 45 and 59 ml/min, "moderate CKD" for a value between 30 and 44 ml/min, and "severe or end-stage CKD" for a value below 30 ml/min), and the comorbidity burden (at least one Cumulative Illness Rating Scale-Geriatric (CIRS-G) grade 3 or 4 comorbidity [27]). Polymedication was defined as taking five or more medications daily.

At inclusion the geriatric assessment included MMSE, memory complaint, iADL score. The thresholds for defining the NCD classes were adapted from Perneczky et al.’s [28,29], (Table 1). The adaptation of the Perneczky criteria, combining MMSE, memory complaints and IADL score allows us to get closer to the most recent definition of neurocognitive disorders, according to the DSM V [30]. Indeed, by using only MMSE (and CDR according to Perneczky et al.), we would only have appreciated the severity of the neurocognitive disorders. The use of the IADL score allows us to document the loss of autonomy, essential to differentiate mild and Major NCD according to the DSM V. Finally the presence of a memory complaint makes it possible to raise awareness of the diagnosis of Mild NCD, in patients with an MMS > = 26/30.

**Table 1:** Classification of cognitive disorders using MMSE, iADL and memory complaint.

	Covariate construction with data		
	MMSE	Memory complaint	iADL score
No NCD	≥ 26 &	No &	0−4
Mild NCD	25−21 or	Yes	& 4/4
Moderate NCD	25−21 or	Yes	& ≤3/4
Major NCD	≤20	Yes or no	& ≤3/4

NCD: neurocognitive disorder; MMSE: Mini Mental State Examination; iADL: Instrumental Activities of Daily Living.

No patient were MMSE ≤ 20 & iADL 4/4.

The primary tumor site was categorized as breast cancer, other gynecological cancer (ovarian, womb and endometrial cancers, etc.), colorectal cancer, other digestive tract cancer (esophageal, stomach and pancreatic cancers, etc.), lung cancer, prostate cancer, other urinary tract cancer (bladder and kidney cancer, etc.), or other cancer. The tumor stage was defined as metastatic or non-metastatic. All the data were collected on a standardized case report form.

### 2.3 Statistical analysis

Analyses were performed using Stata software (version SE 15.0, Stata College Station, TX, USA). All tests were two-tailed, and the threshold for statistical significance was set to p < 0.05.

Baseline socio-demographic, geriatric, cancer related were presented as a function of the patient’s NCD status: no NCD, mild NCD, moderate NCD, and major NCD. Quantitative co-variables were presented as the mean (SD) or (for age) the median [interquartile range (IQR)] and compared by NCD class in Student’s t-test. Pairs of classes were compared in an analysis of variance with Bonferroni’s correction. Qualitative co-variables were described as the number of events per category, the frequency, and the 95% confidence interval (CI). For the comparisons of NCD classes, the overall chi-squared test statistic was reported. We also compared the different NCD classes in pairs and applied Bonferroni’s correction for multiple testing [31].

Survival curves by NCD classes were plotted using the Kaplan-Meier method and compared in a log-rank test. Mortality was also analyzed using semiparametric Cox models with age as a time variable. Proportional hazards were checked with the Schoenfeld residual test and graphically. Multivariable mortality analyses were performed using a manual, forward stepwise technique. We included variables that were associated significantly with NCD and mortality (p-value<0.2) and clinically relevant variables identified in a literature [32]. As we are in an analytical framework aiming to understand the prognosis value of NCD, we provide the most parcimonious multivariable models and not fully-adjusted model. To properly understand the mechanism linking NCD to prognosis, we perform a multivariable model with adjustement for treatment strategy and one without.

We tested several interactions known from the literature: type of cancer and metastasis, NCD and type of treatment, fatigue and ECOG-PS status, NCD and depression [32].

We performed univariable and then multivariable analyses of the treatment strategy (curative, palliative, or exclusive supportive care) by respectively using the chi-squared test for qualitative variables and Student’s t-test for quantitative variables and multinomial logistic regression model.

## 3 Results

Among the eligible patients included in the ELCAPA cohort from January 31, 2007 to December 29, 2017) (n = 3788), we excluded 34 with delirium, 70 who did not speak French, 182 with a hematological cancer, and 718 lacking an MMSE score (846) and an IADL/4 score (332). The final sample size was 2784 patients. The median [IQR] age in the overall study population was 82 [78;86], with majority of women (55%). Most of the patients were outpatients (74.5%). The most frequent cancers were breast, digestive tract and prostate cancers. Fifty-one percent of the cancers were metastatic at diagnosis. After the multidisciplinary team meeting, the final decision was surgery for 26% of the patients, systemic drug therapy for 44%, and radiotherapy for 20%. 49% of the patients received curative treatment, 35% received palliative treatment, and 16% received exclusive supportive care.

Of the population study, 1010 (36%) were free of NCD, 958 (34%) had a mild NCD, 486 (18%) had a moderate NCD, and 330 (12%) had a major NCD (Table 2). Patients with NCD were significantly older than other patients; they were more likely to be inpatients and had poorer general health, having at least one CIRS-G grade 3 or 4 comorbidity. Comorbidities significantly associated with NCD were depression, arterial hypertension, other cardiovascular diseases, and CKD.

**Table 2:** Characteristics of the overall study population and by NCD classes.

Covariate	Whole Population	No NCD N = 1010	Mild NCD N = 958	Moderate NCD N = 486	Major NCD N = 330	p-value
Age n = 2783, years	82 [78 ;86]	81 [77;85]$£	81 [77;85]¤#	83 [80;87]$¤	84 [78;88]£#	0.026
Sex n = 2784 (female)	1523 (55)	516 (51)$	532 (56)	274 (56)	201 (61)$	0.009
Type of consultation n = 2784 (outpatient vs. inpatient)	2073 (74)	800 (79)	744 (78)	324 (67)	205 (62)	<0.001
Living at home n = 2776 (vs. nursing home)	2630 (85)	984 (98)$∼	919 (96)*¤	440 (91)$*¤	287 (87)∼¤	0.005
ADL/6 n = 2766 (abnormal ≤5/6)	707 (26)	133 (13)$*	126 (13)¤£	240 (49)$¤#	208 (63)*£#	<0.001
IADL /4 n = 2628 (abnormal ≤3/4)	1083 (41)	267 (27)$*¤	0#μ¤	486 (100)$#	330 (100)*μ&	<0.001
History of falls n = 2736 (yes)	787 (29)	218 (22)	232 (25)	185 (39)	152 (48)	<0.001
One-leg standing balance test n = 2194 (normal)	1412 (64)	508 (58)$*	487 (58)£¤	257 (84)$£	160 (89)*¤	<0.001
Fatigue n = 2749 (yes)	1961 (71)	675 (67)$	342 (68)£¤	391 (81)£	253 (78)$¤	<0.001
ECOG-PS n = 2764						<0.001
overall health preserved (PS 0−1)	1479 (53)	667 (66)	657 (69)	103 (21)	52 (16)	
reduced physical activity (PS 2)	632 (23)	199 (20)	184 (19)	164 (34)	85 (26)	
bedridden or in a chair > 50% of the time (PS 3−4)	653 (24)	136 (14)	110 (12)	217 (45)	190 (58)	
Nutritional status (% weight loss) n = 2480						<0.001
no weight loss	1395 (56)	558 (61)	509 (60)	192 (45)	136 (49)	
malnutrition	496 (20)	185 (20)	162 (19)	97 (23)	52 (19)	
severe malnutrition	589 (24)	179 (19)	184 (22)	137 (32)	89 (32)	
Mini GdS score n = 2546 (abnormal ≥1)	851 (33)	260 (27)	267 (31)	197 (46)	127 (44)	<0.001
Arterial hypertension n = 2774 (yes)	1880 (68)	671 (66.5)$	625 (65)*	341 (70.5)	243 (75)$*	0.007
Other cardiovascular diseases n = 2666 (yes)	1064 (40)	387 (40)	327 (36)	210 (45)	140 (45)	0.002
≥1 CIRS-G category ≥3 (yes) n = 2657 (yes)	1655 (62)	562 (58)	487 (53)	342 (74)	264 (85)	<0.001
CKD n = 2489						<0.001
no CKD (GFR ≥ 60 ml/min	875 (35)	352 (39)	301 (35)	141 (32)	81 (28)	
mild CKD (GFR 45−59 ml/min)	773 (31)	283 (31)	270 (31)	135 (31)	85 (29)	
moderate CKD (GFR 30–44 ml/min)	623 (25)	204 (23)	227 (26)	99 (23)	93 (32)	
major or end-stage CKD (GFR < 30 ml/min)	218 (9)	64 (7)	60 (7)	62 (14)	62 (14)	
Polymedication n = 2667 (yes)	1796 (67)	61 (63)$*	590 (65)£¤	350 (75)$£	246 (76)*¤	<0.001
Type of cancer n = 2778						
gynecological (other than breast)	170 (6)	77 (8)	58 (6)	19 (4)	16 (5)	<0.001
breast	637 (23)	220 (22)	236 (25)	98 (20)	83 (25)	
digestive tract (other than colorectal)	441 (16)	144 (14)	168 (18)	80 (16)	49 (15)	
colorectal	431 (16)	150 (15)	127 (13)	107 (22)	47 (14)	
lung	191 (7)	77 (8)	53 (6)	32 (7)	29 (9)	
urinary tract (other than prostate)	366 (13)	149 (45)	131 (14)	54 (11)	32 (10)	
prostate	287 (10)	109 (11)	104 (11)	45 (9)	29 (9)	
others	255 (9)	83 (8)	78 (8)	50 (10)	44 (13)	
Metastasis (yes) n=2422	1246 (51)	465 (52)	397 (48)	238 (57)	146 (54)	0.011
Treatment strategy n = 2297						<0.001
Curative	1132 (49)	435 (52)	453 (56)	149 (37)	95 (37)	
Palliative treatment	801 (35)	316 (38)	272 (34)	146 (36)	67 (26)	
Exclusive supportive care	364 (16)	78 (9)	82 (10)	106 (26)	98 (38)	

n = number of available data in whole population.

ECOG-PS: Eastern Cooperative Oncology Group performance status; ADL: Activities of Daily Living, IADL: Instrumental Activities of Daily Living; Mini GDS: Mini Geriatric Depression Scale; CKD: chronic kidney disease. Polymedication was defined as ≥5 drugs daily, %weight loss: percentage of weight lost in 1 or 6 months, according to the HAS classification: malnutrition if >5% in 1 month or >10% in 6 months, severe malnutrition if >10% in 1 month or >15% in 6 months, CIRS-G = Cumulative Illness Rating Scale-Geriatric.

Quantitative covariates are quoted as the mean (SD), and qualitative covariates are quoted as the frequency (percentage).

*p < 0.05 in a univariable analysis by NCD classes, using the chi-squared test for qualitative variables and Student’s t-test for quantitative variables. $£¤*#p < 0.05 in pairwise comparisons of NCD classes, using an ANOVA for qualitative variables and Student’s t-test for quantitative variables, with Bonferroni correction.

**Fig. 1:**
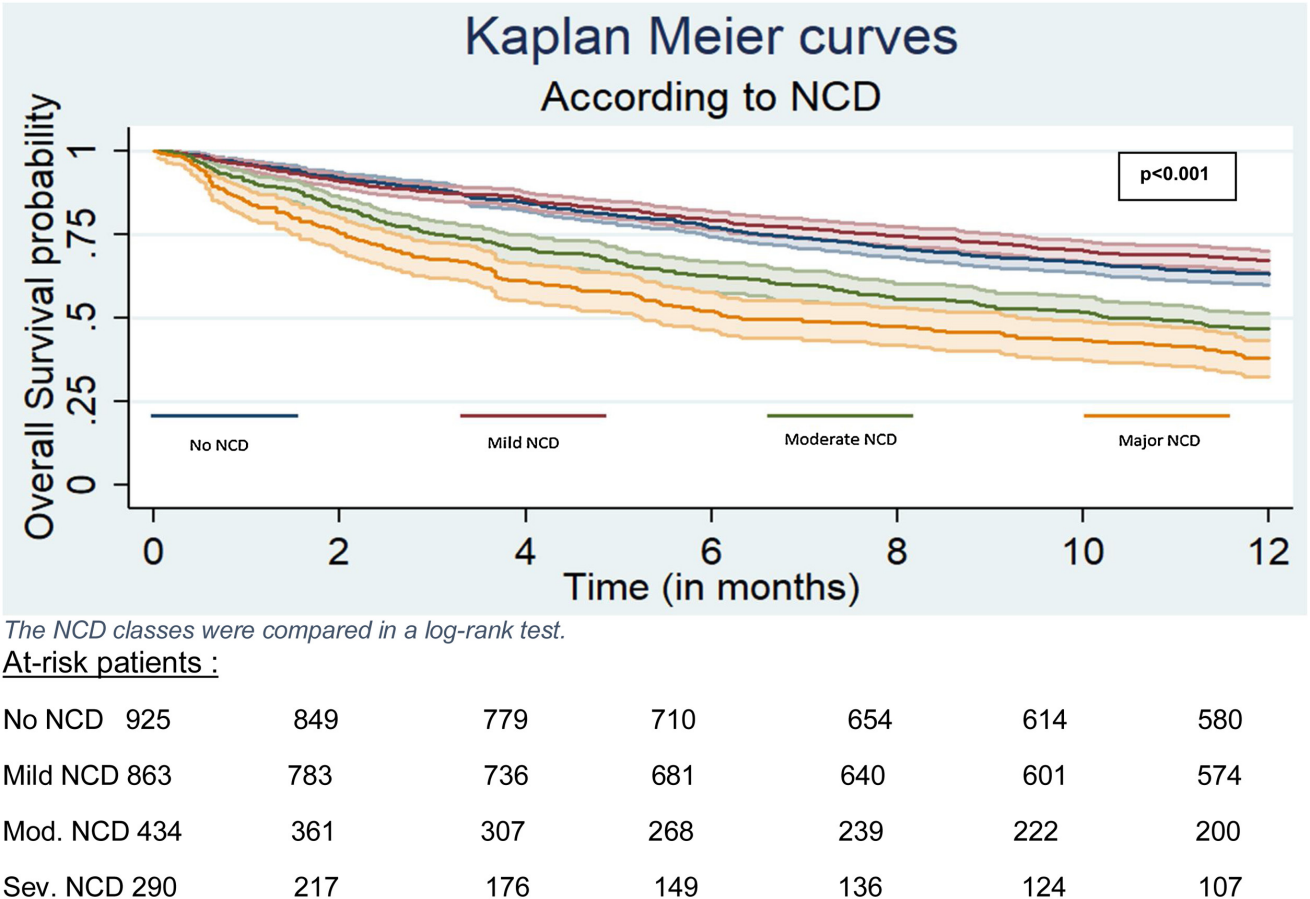
12-month survival in a Kaplan-Meier plot. Abreviations: NCD: neuro-cognitive discorder; mod = moderate; sev = severe

The severity of the NCD was significantly associated with exclusive supportive care decision and inversely associated with curative treatment decision (p < 0.001) (Table 2). Furthermore, the severity of the NCD was associated with higher mortality: the crude hazard ratio (HR) [95%CI] was 1.69 [1.43–2] for moderate NCD vs. no NCD and 2.23 [1.59–1.99] for major NCD vs. no NCD (p < 0.001) (Fig. 1).

In an univariable analysis, male sex, older age, inpatient status, the type of cancer, metastatic status, fatigue, ECOG-PS, the ADL score, the IADL score, weight loss, an abnormal Mini GdS score, another cardiovascular disease, CKD, polymedication, and at least one CIRS-G grade 3−4 co-morbidity were associated with higher mortality at 12 months (Table 3).

**Table 3:** 12-month mortality in a semi-parametric Cox model with age as the time variable.

Variable	Univariable analysis n = 2784				Multivariable analysis n = 1642			
	HR	95%CI		p-value	HRa	95%CI		p-value
NCD (vs. no NCD)				<0.001				0.0008
mild NCD	0.88	0.75	1.03		0.93	0.76	1.12	0.428
moderate NCD	1.69	1.43	2.00		0.98	0.79	1.22	0.854
major NCD	2.23	1.86	2.67		1.54	1.19	1.98	0.001
Female (vs. male)	0.65	0.57	0.73	<0.001				
Inpatient (vs. outpatient)	2.81	2.48	3.19	<0.001	1.55	1.32	1.82	<0.001
Living at home (vs. nursing home)	0.87	0.67	1.14	0.317				
Abnormal ADL score	2.52	2.22	2.85	<0.001				
Abnormal IADL4 score	2.42	2.13	2.75	<0.001				
History of falls (yes)	1.36	1.20	1.55	<0.001				
One-leg standing balance test <5 s (yes)	2.02	1.70	2.39	<0.001				
Fatigue (yes)	2.21	1.89	2.60	<0.001	1.27	1.03	1.56	0.027
ECOG-PS (vs. PS 0−1)				<0.001				<0.001
reduced physical activity (PS 2)	2.11	1.80	2.48		1.37	1.12	1.68	0.003
bedridden or in a chair >50% of time (PS 3-4)	4.24	3.68	4.90		2.53	2.04	3.14	<0.001
% weight loss (vs. no weight loss)				<0.001				0.0349
malnutrition	1.83	1.54	2.16		1.05	0.86	1.28	
severe malnutrition	2.73	2.35	3.16		1.26	1.05	1.52	
Abnormal Mini GdS score (yes)	1.71	1.50	1.95	<0.001				
Arterial hypertension (yes)	1.06	0.93	1.22	0.354				
Other cardiovascular disease (yes)	1.27	1.12	1.44	<0.001				
CKD (vs. no CKD)				0.0022				
mild CKD (GFR 45−59 ml/min)	1.04	0.89	1.23					
moderate CKD (GFR 30–44 ml/min)	1.20	1.01	1.41					
major or end-stage CKD (GFR < 30 ml/min)	1.54	1.24	1.91					
≥1 CIRS-G category ≥3 (yes)	1.93	1.68	2.22	<0.001				
Polymedication	1.38	1.20	1.59	<0.001				
Type of cancer (vs. breast)				<0.001				<0.001
gynecological (other than breast)	0.71	0.53	0.96		2.10	1.35	3.27	0.001
digestive tract (other than colorectal)	2.15	1.87	2.48		4.72	3.40	6.55	<0.001
colorectal	0.77	0.64	0.92		1.77	1.25	2.51	0.001
lung	2.04	1.68	2.49		4.93	3.40	7.14	<0.001
urinary tract (other than prostate)	1.26	1.07	1.49		3.89	2.77	5.47	<0.001
prostate	0.71	0.57	0.88		1.86	1.28	2.69	0.001
others	1.85	1.55	2.22		3.56	2.49	5.08	<0.001
Metastasis (yes)	3.24	2.81	3.75					<0.001
Type of treatment (vs. curative)				<0.001				<0.001
palliative treatment	3.61	3.04	4.27		1.48	1.18	1.86	0.001
exclusive supportive care	7.05	5.86	8.49		2.53	1.99	3.23	<0.001

ECOG-PS: Eastern Cooperative Oncology Group performance status; ADL: activities of daily living; IADL: Instrumental Activities of Daily Living; Mini GDS: Mini Geriatric Depression Scale; CKD: chronic kidney disease. Polymedication was defined as ≥5 drugs daily. % weight loss was measured as the percentage of weight loss at 1 or 6 months according to the HAS definition (malnutrition if >5% in 1 month or >10% in 6 months and severe malnutrition if >10% in 1 month or >15% in 6 months).

Analysis of mortality at 12 months in univariable and multivariable Cox semi-parametric models with age as the time variable. Adjustment variables were: inpatient, fatigue, ECOG-PS, %weight loss, type of cancer, type of treatment.

In a multivariable survival analysis, the factors significantly associated with 12-month mortality were the type of cancer (all types of cancer vs. breast cancer), metastatic status, inpatient status, fatigue, poor ECOG-PS, weight loss, treatment strategy, and NCD (Table 2**).** Only the major NCD class was independently associated with higher mortality (HR [95%CI] = 1.54 [1.19–1.98], p < 0.001). In a multivariable analysis not adjusted for treatment strategy but adjusted for the other variables, the HR [95%CI] for a major NCD was 1.78 [1.39–2.29] (p < 0.001).

Factors significantly associated with the therapeutic strategy in the univariable analysis are presented in Table 3.

**Fig. 2:**
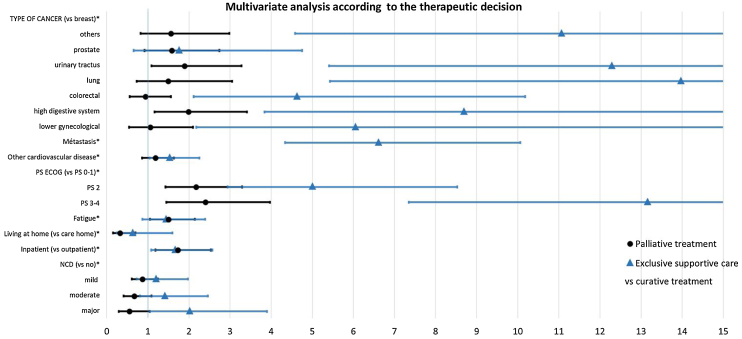
Multivariable analysis (multinomial logistic regression model according to the treatment strategy). ECOG-PS: Eastern Cooperative Oncology Group performance status; ADL: activities of daily living; Mini GDS: Mini Geriatric Depression Scale. CRP: C-reactive protein; CKD: chronic kidney disease. Polymedication was defined as ≥5 drugs daily. Multinomial logistic regression model. *p < 0.05 **p < 0.001: degrees of significance

The factors independently associated with exclusive supportive care (vs. curative treatment) in a multivariable analysis were major NCD, inpatient status the type of cancer, metastatic status, fatigue, poor ECOG-PS, living at home (vs. a nursing home), an abnormal Mini GdS score, and cardiovascular disease (Table 4 and Fig. 2).

**Table 4:** Univariable analysis according to the type of treatment decision made in the multidisciplinary team meeting.

Variable	Univariable analysis n = 2297				
	Curative treatment	Palliative treatment	Exclusive supportive care	Whole sample	p-value
Sex (female)	719 (64)	393 (49)	176 (48)	1288 (56)	<0.001
Type of consultation (outpatient vs. inpatient)	166 (15)	264 (33)	175 (48)	605 (26)	<0.001
Living at home (vs. nursing home)	1083 (95)	757 (5)	335 (7)	1083 (95)	0.035
ADL score (abnormal ≤5/6) (yes)	180 (16)	205 (26)	203 (56)	588 (26)	<0.001
IADL4 score(abnormal ≤3/4) (yes)	316 (29)	320 (42)	239 (74)	875	<0.001
History of falls (yes)	268 (24)	221 (28)	148 (42)	637 (28)	<0.001
One-leg standing balance test (normal)	551 (58)	427 (65)	178 (84)	1156 (64)	<0.001
Fatigue (yes)	701 (62)	615 (78)	303 (83)	1619 (71)	<0.01
ECOG-PS					<0.001
overall health preserved (PS 0−1)	795 (71)	378 (47)	60 (17)	1233 (54)	
reduced physical activity (PS 2)	188 (17)	228 (29)	91 (25)	507 (22)	
bedridden or in a chair >50% of the time (PS 3−4)	140 (12)	191 (24)	211 (58)	542 (24)	
Nutritional status (% weight loss)					<0.001
No weight loss	690 (69)	345 (47)	121 (38)	1156 (56)	
malnutrition	171 (17)	171 (24)	70 (22)	412 (20)	
severe malnutrition	139 (14)	211 (30)	131 (41)	481 (23)	
Abnormal Mini GdS score (yes)	293 (28)	245 (34)	159 (49)	697 (33)	<0.001
Arterial hypertension (yes)	746 (66)	551 (69)	248 (69)	1545 (67)	0.307
Other cardiovascular disease (yes)	400 (63)	313 (59)	181 (49)	894 (60)	<0.001
≥1 CIRS-G category ≥3 (yes)	554 (51)	505 (66)	283 (81)	1342 (61)	<0.001
Polymedication (yes)	690 (64)	533 (31)	260 (26)	1483 (32)	0.001
Type of cancer					<0.001
gynecological (other than breast)	79 (7)	50 (6)	19 (5)	148 (6)	
breast	398 (35)	147 (18)	18 (5)	563 (25)	
digestive tract (other than colorectal)	137 (12)	137 (17)	75 (21)	349 (15)	
colorectal	168 (15)	113 (14)	75 (21)	356 (16)	
lung	44 (4)	62 (8)	43 (12)	149 (7)	
urinary tract (other than prostate)	134 (12)	89 (11)	68 (19)	291 (13)	
prostate	101 (9)	114 (14)	12 (3)	227 (10	
others	69 (6)	88 (11)	52 (14)	209 (9)	
Metastatic status (yes)	173 (18)	669 (89)	198 (65)	104 (51)	<0.001

n = number of available data in whole population.

ECOG-PS: Eastern Cooperative Oncology Group performance status. ADL: activities of daily living; IADL: Instrumental Activities of Daily Living; Mini GDS: Mini Geriatric Depression Scale; CRP: C-reactive protein. BMI: body mass index. Polymedication was defined as ≥5 drugs daily. CKD: chronic kidney disease. n: number of available data. % weight loss: measured as the percentage weight loss at 1 or 6 months according to the HAS definition (malnutrition if >5% in 1 month or >10% in 6 months and severe malnutrition if >10% in 1 month or >15% in 6 months).

Quantitative covariates are quoted as the mean (SD) and qualitative covariates are quoted as the frequency (percentage).

*p < 0.05 in a univariable analysis using the chi-squared test for qualitative variables and Student’s t-test for quantitative variables.

## 4 Discussion

### 4.1 Main results

In our longitudinal, observational study of a cohort of 2784 older adults (median age: 82) with a solid cancer, referred to a geriatrician prior to treatment initiation, 36% were free of NCD, 34% had a mild NCD, 17% had a moderate NCD, and 13% had a major NCD. Patients with a major NCD had an increased risk of death within 12 months, independently of the type of cancer, metastatic status, the initial mode of care (inpatient vs. outpatient treatment), overall condition (as assessed by the ECOG-PS and fatigue), and weight loss. Additional adjustment for the treatment strategy only slightly weakened the relationship between a major NCD and death. Moderate and major NCDs were independently associated with palliative treatment after we took into account of the following confounding factors: inpatients, the type of cancer, metastatic stage, fatigue, ECOG-PS, the type of residence, depression, and cardiovascular disease.

### 4.2 External validity

Our study population was representative of older adults with cancer, with a median age of 82, a and various comorbidities [33,34]. The prevalence of NCD in our study (64%) was higher than in other studies. This might be due to our particular definition of NCD and that all of our patients had been referred for a geriatric assessment, which was often prompted by frailty factors like cognitive impairment.

We found that major NCD was independently associated with higher 12-month mortality in our population and that the association was not explained fully by the identified confounding factors (general health status, nutritional status, the type of cancer, metastatic status, and the treatment strategy). Our results are in line with most of the literature data on this topic (namely that the presence of a major NCD has independent prognostic value for higher mortality [19,35]) recorded in older patients without cancer. We found very few studies of the prognostic value of NCD in older adults with cancer. Aldricks and al. (2013) [36,37] reported on a prospective cohort of 143 patients with colorectal cancer and 55 patients with breast cancer. In the open-label prospective trial of 111 patients with ovarian cancer by Falandry et al. (2013) [38]. In 2013, Hamaker et al. reported on a multicenter randomized study of the safety and efficacy of first-line, single-agent palliative chemotherapy for 732 metastatic breast cancer patients [39]. Soubeyran et al. (2012) prospectively studied a multicenter cohort of 348 patients with a solid or hematological cancer [40]. These studies found an association between NCD (defined by MMSE score ≤24, 25 or 23 respectively) and higher mortality in univariable analysis but not in a multivariable analysis. Compared to our study they might have lacked statistical power due to the small sample size, the small percentage of patients with NCD partly explain by low median age (between 75 and 79). However, In a retrospective cohort study of 106 061 patients aged 68 years or older diagnosed as having breast, colon, or prostate cancer, using data from the linked Surveillance, Epidemiology and End Results– Medicare, Raji MA et al. assessed the risks of mortality from cancer and noncancer causes, stratified by presence or absence of preexisting dementia diagnoses. They found that preexisting dementia diagnoses were associated with high mortality, mostly from noncancer causes [21].

Adjustment for the treatment strategy (in addition to all the other adjusting variables) did not greatly modify the strength of the association between major NCD and mortality. This finding suggests that NCD is associated to mortality by itself rather than by its consequences on treatment strategy. A recent systematic review found that cognitive impairment in over-65 adults on chemotherapy was associated with higher rates of treatment toxicity, higher mortality and lower treatment rates [41]. However, the review emphasized the following limitations of the studies considered: a geriatric assessment after the choice of treatment, a small sample size, and the exclusion of major NCDs.

Of the others factors associated with 12-month mortality, the general state of health (notably as assessed by the ECOG-PS) is a well-known prognostic factor in general and geriatric oncology [[42], [43], [44]]. It is noteworthy that fatigue (although self-reported and less precise than the ECOG-PS) was found to be an independent prognostic factor. This result is consistent with the recent literature [45,46]. Autonomy (the ADL score), polymedication, and the comorbidity burden (CIRS-G) were associated with mortality in univariate analysis but not in multivariate analysis. This difference might have been due to strong correlations between these variables and the ECOG-PS and the IADL score.

### 4.3 Internal validity

Our study had some limitations. We lacked data on the type of cancer treatment, the protocols, the dose, the adherence and the adverse events. This might have led to overestimation of the strength of the relationship between NCD and mortality. In addition, this does not allow us to differentiate between poorly tolerated chemotherapy with a poor response, and targeted therapy with better tolerance and a significant response, depending on the type of cancer. Thus, we do not explore the benefit/risk ratio of treatment in patients with major NCD. Moreover, we have a substantial number of missing data notably regarding treatment intent and metastasis status leading to a potential selection bias in the final multivariable model.

The study had a number of strengths. Firstly, the ELPACA study is one of the largest yet prospective cohort studies of older patients with a solid cancer. The way of selection of adjustement variables based on known confounders in the literature. And addressing potential issues like multicollinearity ensure the robustness of the final model. We decided to classify the NCD on the basis of the MMSE test result because this screening test has been validated in routine practice for the detection of NCD [47], easy to use in everyday practice, even though it is imperfect in terms of the threshold for detecting cognitive impairment.Thirdly, our NCD classification was as close as possible to two international classifications that are used in routine clinical practice, which means that results might be extendable to other countries and healthcare systems. Fourthly, we used age as a time variable, in order to mitigate the influence of this potential confounding factor.

### 4.4 Implications

The first implication of our study is to better stratify NCD status of older patients with cancer by taken into account the person’s autonomy (the IADL score and/or the DSM5 definition) and not only the presence of NCD according to the MMSE score). We recommended informing patients and their caregivers that major NCD, in the context of cancer, is a prognosis factor by itself independently from the treatment decision and cancer itself.

The second implication is that our results raise questions about the appropriate level of care for cancer in the context of pre-existing moderate and severe NCDs. These complex issues will require further studies including how the presence of NCD affects treatment decision making, treatment adaptation, treatment-related toxicities, monitoring during treatment, and use of palliative care. Such studies could contribute to the development of guidelines for cancer treatment that take into account the quality of life and life expectancy of patients with NCD.

## 5 Conclusions

Our study’s results suggest that major NCD is an independent prognostic factor for 12-month mortality in older patients with cancer, beyond the cancer type and stage and the cancer treatment strategy.

## Declaration of sources of funding

The ELCAPA study was funded by the 10.13039/501100006364French National Cancer Institute (Institut National du Cancer, INCa), Canceropôle Ile-de-France, Gerontopôle Ile-de-France (Gerond'If for Catherine Conti master’s year), and the Curie Institute, none of which had any role in the design and conduct of the study, the collection, management, analysis, and interpretation of the data, the preparation, review and approval of the manuscript or the decision to submit the manuscript for publication.

## Conflicts of interest

None.
